# Evaluation of genetic isolation within an island flora reveals unusually widespread local adaptation and supports sympatric speciation

**DOI:** 10.1098/rstb.2013.0342

**Published:** 2014-08-05

**Authors:** Alexander S. T. Papadopulos, Maria Kaye, Céline Devaux, Helen Hipperson, Jackie Lighten, Luke T. Dunning, Ian Hutton, William J. Baker, Roger K. Butlin, Vincent Savolainen

**Affiliations:** 1 Grand Challenges in Ecosystem and the Environment Initiative, Imperial College London, Silwood Park Campus, Ascot, Berkshire SL5 7PY, UK; 2 School of Biological Sciences, University of Aberdeen, Aberdeen AB24 3FX, UK; 3 Institut des Sciences de l'Evolution de Montpellier, UMR 5554, 34095 Montpellier, France; 4 Department of Biology, Dalhousie University, Halifax, Nova Scotia, Canada B3H 4R2; 5 Lord Howe Island Museum, Lord Howe Island, PO Box 157, New South Wales 2898, Australia; 6 Royal Botanic Gardens, Kew, Richmond, Surrey TW9 3DS, UK; 7 Department of Animal and Plant Sciences, University of Sheffield, Sheffield S10 2TN, UK

**Keywords:** local adaptation, ecological speciation, genetic structure

## Abstract

It is now recognized that speciation can proceed even when divergent natural selection is opposed by gene flow. Understanding the extent to which environmental gradients and geographical distance can limit gene flow within species can shed light on the relative roles of selection and dispersal limitation during the early stages of population divergence and speciation. On the remote Lord Howe Island (Australia), ecological speciation with gene flow is thought to have taken place in several plant genera. The aim of this study was to establish the contributions of isolation by environment (IBE) and isolation by community (IBC) to the genetic structure of 19 plant species, from a number of distantly related families, which have been subjected to similar environmental pressures over comparable time scales. We applied an individual-based, multivariate, model averaging approach to quantify IBE and IBC, while controlling for isolation by distance (IBD). Our analyses demonstrated that all species experienced some degree of ecologically driven isolation, whereas only 12 of 19 species were subjected to IBD. The prevalence of IBE within these plant species indicates that divergent selection in plants frequently produces local adaptation and supports hypotheses that ecological divergence can drive speciation in sympatry.

## 1. Introduction

The role of natural selection in speciation has received renewed interest owing to the growing body of data that has demonstrated the potential for divergent selection to overcome the homogenizing effects of gene flow between populations [1–6]. A number of classic examples of ecological speciation—where the evolution of reproductive isolation between populations ultimately stems from divergent selection—have emerged [1,2,7]. However, there is still debate as to the extent to which ecologically driven isolation (e.g. via selection against hybrids and migrants) or geographically driven isolation (as a result of dispersal limitation) is the most significant component of speciation [8–10]. The tendency for geographically separated populations and individuals to be less likely to reproduce with each other is manifested as a pattern of isolation by distance (IBD; [11]). IBD is often quantified as the correlation between increasing neutral genetic divergence and increasing geographical distance [12]. Alternatively, during ecological speciation, environmental and ecological differences between populations can constrain reproduction and migration. This can occur as a result of selection against maladapted individuals and alleles, which, in turn, reduces gene flow. This process may lead to local adaptation to particular environments and eventually to speciation [1,6,13]. Selection against migrants and hybrids can yield a pattern similar to that stemming from IBD, where increasing environmental dissimilarity between locations is correlated with neutral genetic divergence between populations or individuals [14]. This pattern of isolation by environment (IBE) is increasingly seen as a signature or precursor to incipient ecological speciation [13,15].

In reality, geographical and ecological processes that can influence spatial genetic patterns are not mutually exclusive, and decreased migration and reproduction can stem from both sources simultaneously. Thus, attempts to establish the contributions of geographical and ecological mechanisms to isolation in natural populations are complicated by the need to disentangle the effects from one another [13–18]. Isolation driven by competition with other species in the local community may also be an important component of population divergence. Such isolation by community (IBC) is often neglected in studies of IBE, which concentrate on the abiotic environment, but it is a powerful driver of ecological displacement [19] and potentially of local adaptation. Additionally, patterns of gene flow can arise where migration is not dependent on the measured environmental gradient, but the movement of migrants is determined by additional factors, such as wind direction [18]. This can lead to counter-gradient isolation where gene flow is higher between dissimilar environments, further complicating observed patterns.

Many studies have examined only IBD or IBE, but a growing number of different approaches have been exploited in order to quantify IBD and IBE at the same time (see [18,20] for reviews of these approaches). Two recent studies have examined patterns of IBD and IBE in a wide range of taxa [15,18], revealing that IBE may be a common phenomenon. A mixed effects meta-analysis of 106 studies found that environmental differences accounted for 1.3–3.7% of neutral genetic divergence when IBD was taken into account and 2.4–4.3% when it was not [15]. A different study found that 52 of 70 studies provided evidence of IBE or both IBE and IBD [18]. Frequently, studies have assessed IBE for one or a few environmental gradients, or have summarized contribution of many environmental variables into a single measure of environmental distance [16]. Although Mantel tests and AMOVAs have been a mainstay of landscape genetics and assessments of IBD, IBE and isolation by adaptation [13,15–18,21–23], multivariate approaches (e.g. multiple matrix regression [16]) have received increasing interest as they hold great potential for determining how geographical distance, phenotypic dissimilarity and multiple landscape barriers can interact to alter patterns of relatedness [16–18,20]. Here, we exploit an extension of the multiple matrix regression approach [16] by including a model averaging procedure [24]. Model averaging provides the advantage that uncertainty in parameter estimates due to model selection can be taken into account, reducing the risk of false-positives [24,25]. This approach also permits comparison of analyses that use a single, unified measure of environmental dissimilarity with regressions of multiple variables.

Lord Howe Island (LHI) provides a unique opportunity to determine the prevalence of IBE in a single location across a range of phylogenetically closely and distantly related taxa that have been subjected to broadly similar environmental factors on a geologically similar time scale [8,26–28]. The product of a volcanic eruption 6.9 Ma, the tiny LHI is located 600 km from the nearest land mass (Australia). The island is highly heterogeneous with a mixture of geological formations (both basaltic and calcarenite) and a range of habitat types, ranging from sclerophyllous temperate rainforest in the lowlands to moist cloud forest at the summit of Mt Gower (875 m), making it home to 90 endemic species [8,27,29]. Studying IBD, IBE and IBC in trees and bushes has a number of advantages, including their static lifestyles, high dispersal abilities and propensity for local adaptation [20], and is particularly relevant to the flora of LHI. Recent studies have shown that ecological speciation with gene flow is likely to have taken place in multiple genera, and as much as 8.2% of the flora may have been the product of speciation that has taken place within the confines of the island [8,10,26,28,30]. Previous research has provided evidence that local adaptation has driven diversification in several of these genera (including *Coprosma*, *Metrosideros* and *Howea* [26,28]). IBD and IBE had weak but significant effects in several species, but only altitude and soil pH were tested as explanatory variables of genetic variation [26,30].

It is unclear whether the speciation and adaptation seen in *Coprosma*, *Metrosideros* and *Howea* is peculiar to these genera, or whether IBE is common throughout the LHI flora. The goal of this study was to quantify the contributions of IBD, IBE and IBC to genetic relatedness in 19 species. These include species that are thought to be the products of sympatric speciation events as well as those that have colonized LHI and evolved allopatrically from their parent populations [8]. We also assess whether IBD, IBE and IBC have led to the emergence of distinct genetic clusters within any of the endemic LHI species, which may represent the early stages of the speciation continuum.

## 2. Material and methods

### (a) Study species and data collection

Genetic and ecological data were collected for individuals from 19 species for this study; 18 are endemic to LHI and one is a non-endemic, native species (table 1). Two species (*Coprosma* sp. nov. and *C. putida*-S) have not been formally described, but both are genetically and morphologically distinct from other *Coprosma* populations [8,26] and are treated as discrete species here. Previous research [8,26,27] suggests that nine of the endemic species (from the genera *Coprosma*, *Metrosideros* and *Howea*) are the products of speciation with gene flow that has occurred on LHI (referred to as the ‘sympatric speciation group’, SSG). These within-island speciation events can be considered as sympatric under the biogeographic definition that we use here; however, under strict population genetic definitions, these events may be considered as parapatric speciation [26,30,31]. Population genetic markers (amplified fragment length polymorphisms, AFLPs [32]) and ecological data have been generated for the SSG species previously [26], and these data were combined with new data for a further 10 species for this study. These data are composed of AFLP genotypes and environmental data for 10 variables collected in the field or extracted from GIS layers for each individual specimen (see [26] for materials and methods).

**Table 1.** Characteristics of study species.

genus	species	speciation group	endemic	seed dispersal	pollination	typical habit	*n* individuals	*n* loci
*Alyxia*	*lindii*	allopatric	yes	animal	animal	scrambling climber	29	82
*Alyxia*	*ruscifolia*	allopatric	no	animal	animal	shrub	31	60
*Atractocarpus*	*stipularis*	allopatric	yes	animal	animal	small tree	46	223
*Coprosma*	*prisca*	allopatric	yes	animal	wind	shrub	49	104
*Dracophyllum*	*fitzgeraldii*	allopatric	yes	wind	animal	tree	28	144
*Geniostoma*	*petiolosum*	allopatric	yes	animal	animal	small tree	31	67
*Macropiper*	*excelsum* subsp. *psittacorum*	allopatric	yes	animal	wind	shrub	33	175
*Macropiper*	*hooglandii*	allopatric	yes	animal	wind	shrub	27	147
*Xylosma*	*maidenii*	allopatric	yes	animal	wind	small tree	41	164
*Zygogynum*	*howeanum*	allopatric	yes	animal	animal	small tree	43	394
*Coprosma*	*huttoniana*	sympatric	yes	animal	wind	shrub to small tree	43	819
*Coprosma*	*lanceolaris*	sympatric	yes	animal	wind	shrub	102	819
*Coprosma*	*putida*-N	sympatric	yes	animal	wind	shrub to small tree	111	819
*Coprosma*	*putida*-S	sympatric	yes	animal	wind	small tree	26	819
*Coprosma*	sp.nov	sympatric	yes	animal	wind	scrambling shrub	15	819
*Howea*	*belmoreana*	sympatric	yes	wind	animal	tree	161	900
*Howea*	*forsteriana*	sympatric	yes	wind	animal	tree	188	900
*Metrosideros*	*nervulosa*	sympatric	yes	animal	wind	shrub to small tree	78	478
*Metrosideros*	*sclerocarpa*	sympatric	yes	animal	wind	tree	72	478

The remaining nine endemic taxa are most likely to have evolved anagenetically following colonization of the island [8]. The LHI population of the non-endemic species *Alyxia ruscifolia* differs in leaf morphology from the Australian population, but it has not been described as a distinct subspecies [33]. These 10 species are subsequently referred to as the ‘allopatric speciation group’ (ASG). General features of the ecology of these species are listed in table 1. For DNA analyses, leaf tissue was collected from mature individuals of these species on LHI and dried using silica gel. For consistency with the previous study [26], the altitude and geographical position of each sample were recorded using an eTrex summit HC GPS with a built-in barometric altimeter. For the remaining nine variables, data for each specimen were extracted from the 10 × 10 m raster grids of Papadopulos *et al*. [26] based on GPS location. Variables included were; Euclidean distance to the nearest creek, Euclidean distance to the coast (a proxy for salt deposition), available light, soil water, soil pH, aspect of the slope, gradient of the slope and vector ruggedness (a measure of topographic heterogeneity [34]). The predominant winds on LHI come from the northeast [29]. To reflect this, aspect of the slope was converted into a continuous measure of northeasterly wind exposure ranging from 0 (equivalent to a southwest aspect) to 180 (i.e. a northeast aspect) [26].

### (b) Genotyping

A method modified from the ‘CTAB’ protocol [35] was used to extract total genomic DNA from 0.3 to 0.5 g of dried leaf material [36]. DNA was purified using DNeasy mini spin columns (Qiagen, Crawley, West Sussex, UK) according to the manufacturer's protocol and subsequently quantified using a Nanodrop (ThermoScientific, Denver, CO). AFLP profiles were generated for each sample as in [26]. Primer trials for 28 primer combinations were carried out on five individuals from each species. Three or four combinations for each species were chosen for the full analysis, based on the number of fragments and polymorphic loci (electronic supplementary material, appendix S1), with the exception of *Coprosma prisca* which was genotyped with the same primer combinations as its congeneric species (see [26]). The raw data were analysed with Genemapper V4 software (Applied Biosystems). Loci (bins) were defined by eye in the range of 50–500 base pairs. Presence/absence at each locus was scored automatically by Genemapper, and scoring was subsequently confirmed manually. Only fragments with signal intensity greater than 50 relative fluorescence units were scored as present. Samples were processed blindly, using extraction codes, to avoid subjectivity in peak scoring. Five individuals of each species were selected at random, re-extracted and genotyped. For these individuals, the two replicates were compared to identify mismatch errors between the genotypes. Loci with more than one mismatch error across the replicates were removed from further analyses.

### (c) Multi-model inference of isolation by distance, isolation by environment and isolation by community

We applied a model averaging approach to determine potential causes of genetic isolation between individuals within each species. This is an extension of the multiple matrix regression approach of Wang [16]. In this procedure, linear regression of a response distance matrix (here, genetic relatedness) on two or more explanatory distance matrices is performed, while controlling interactions among predictor variables. The correlation of each explanatory variable with the response variable was evaluated using model averaging, as described in Burnham & Anderson [24] and implemented by the *MuMIn* package in R [37]. For each species, parameter estimates for submodels (comprising all possible combinations of the included predictor variables) were calculated. Coefficients (effect sizes), unconditional standard errors and 95% confidence intervals for each predictor were calculated by averaging the estimates from submodels in which each term appears and weighting values according to the submodels’ Akaike information criterion (AICc) [24]. This approach has the added benefit that the uncertainty in parameter estimates is known and it avoids pitfalls associated with model selection and statistical null hypothesis testing [24,25]. Parameter estimates were considered significant when the 95% confidence interval did not span zero [25]. In the context of this study, negative effects of explanatory variables with kinship indicate that increasing geographical, environmental or community dissimilarity is correlated with decreasing genetic relatedness. A positive effect denotes a counter-gradient correlation, i.e. individuals are more genetically related in dissimilar environments. To account for the distance matrix nature of our data, the analyses were repeated with 1000 permutations of the kinship matrix. The *z*-scores for variable coefficient estimates from the original analysis were calculated and compared with those stemming from the permutation analyses to ensure that the patterns observed were not random. The resulting *p*-values were used to control the false-discovery rate to 0.05 using *fdrtool* [38].

For each species, a genetic distance matrix composed of pairwise kinship coefficients was calculated using SPAGeDi 1.3 [39] assuming Hardy–Weinberg equilibrium. Within each species, latitudinal and longitudinal coordinates for each individual were converted into a geographical distance matrix using *aflpdat* [40]. This method was preferred over the use of least cost path distances, which are increasingly used in animal studies [16,17], as there is no available information for the LHI plants to suggest that different habitats incur different dispersal limitations for pollen or diaspores. Unless otherwise stated, all further analyses were performed in R [37], distance measures were calculated using the *vegdist* function in the *vegan* package. To construct a measure of the difference in local community composition between individuals, we extracted the vegetative association [29] of each individual from a 10 × 10 m raster grid. Pickard described the most common species present in each of his vegetative associations [29]. Using this information, we constructed a presence/absence matrix for each genus describing which species were likely to co-occur with each specimen. This was then converted into a pairwise Jaccard dissimilarity matrix to describe the distance between individuals in the composition of their local community. We evaluated the environmental variables in two ways. First, we performed a principal component analysis using the *prcomp* function. The resulting scores for each specimen we used to calculate a pairwise Euclidean distance matrix describing environmental dissimilarity between specimens. Second, we calculated Euclidean distance matrices for each environmental variable separately.

Two sets of model averaging analyses were performed for each species to determine the effect of combining environmental variables into a single distance measure (as in [16]) versus assessing the specific effects of each variable: (i) a three matrix analysis was performed using geographical distance (IBD), community dissimilarity (IBC) and the combined environmental dissimilarity matrices (IBE) as explanatory variables, with kinship as the response variable; and (ii) a 12 matrix analysis was performed using geographical distance (IBD), community dissimilarity (IBC) and all 10 of the environmental dissimilarity matrices (IBE) as explanatory variables. Variables were standardized prior to analysis, and collinearity between environmental variables was assessed by calculation of variance inflation factors using the *vif* function in the *car* package. For each species, variables with variance inflation factors of greater than five were removed from the analysis [41].

### (d) Analysis of population structure

To determine whether distinct genetic clusters were present in each species, the presence/absence AFLP data for the ASG species were analysed using the individual-based Bayesian clustering approach implemented in Structure v. 2.3.3 [42], adapted for use with dominant markers [43]. Structure analyses for the SSG species have been performed previously [26]. For this study, all analyses were run using the admixture model with correlated allele frequencies and no *a priori* information of species/population membership. After preliminary runs, analyses of each dataset were conducted with *K* = 1–8 clusters. For each value of *K*, 10 replicates of 80 000 Markov chain Monte Carlo iterations were run, and the first 10 000 iterations of each chain were discarded. Two assessments were used to infer the number of genetic clusters: (i) a comparison of the log probability of the data (*X*) given *K* Ln[Pr(*X*|*K*)] for different values of *K*; and (ii) Δ*K*, the second-order rate of change in Ln[Pr(*X*|*K*)] [44]. Each species was analysed separately, with the exception of the two *Macropiper* species which were analysed together to detect interspecific hybridization between these close relatives.

## 3. Results

### (a) Three matrix analyses

**Figure 1.**
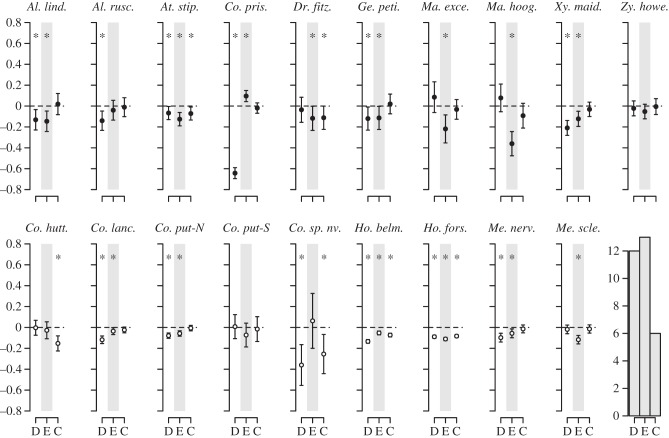
Three matrix model averaging results of IBD (D), IBE (E) and IBC (C) for 19 plant species on LHI. Each plot depicts effect sizes (points) and 95% confidence intervals (error bars) for each parameter. Effect sizes below zero denote negative correlations between predictor variables and relatedness. Effect sizes above zero indicate counter-gradient effects. Estimates were determined as significant (asterisk) when the 95% CI did not span zero and by permutation tests (*α* = 0.045, FDR = 0.05). The bar plot indicates the number of negative effects detected for each parameter across all species. ASG, filled circles, SSG, open circles. *Al. lind., Alyxia lindii; Al. rusc., Alyxia ruscifolia; At. stip., Atractocarpus stipularis; Co. prisc., Coprosma prisca; Dr. fitz., Dracopyllum fitzgeraldii; Ge. peti., Geniostoma petiolosum; Ma. exce., Macropiper excelsum* subsp. *psittacorum; Ma. hoog., Macropiper hooglandii; Xy. maid., Xylosma maidenii; Zy. howe., Zygogynum howeanum; Co. hutt., Coprosma huttoniana; Co. lanc, Coprosma lanceolaris; Co. put-N, Coprosma putida-*N; *Co. put-S, Coprosma putida*-S; *Co.* sp. nov., *Coprosma* sp. nov*.; Ho. belm., Howea belmoreana; Ho. fors., Howea forsteriana; Me. nerv., Metrosideros nervulosa; Me. scle., Metrosideros sclerocarpa*.

When environmental dissimilarity was amalgamated into a single distance measure, significant IBD, IBE and/or IBC were detected in 17 species, but no effects were present in *Zygogynum howeanum* and *Coproma putida*-S (figure 1). The analysis revealed significant IBD in 12 species (six from each group), IBE in 13 species (eight ASG versus six SSG) and IBC in six species (two ASG versus four SSG). IBD was the sole driver of genetic structure in one species (*A. ruscifolia*), as was IBC (in *Coprosma huttoniana*). IBE was the only effect observed in three species. Both IBD and IBE were detected in nine species, and three of these species were subjected to all three modes of isolation. *C. prisca*, was also subjected to IBD and counter-gradient effects of environmental dissimilarity.

### (b) Twelve matrix analyses

**Figure 2.**
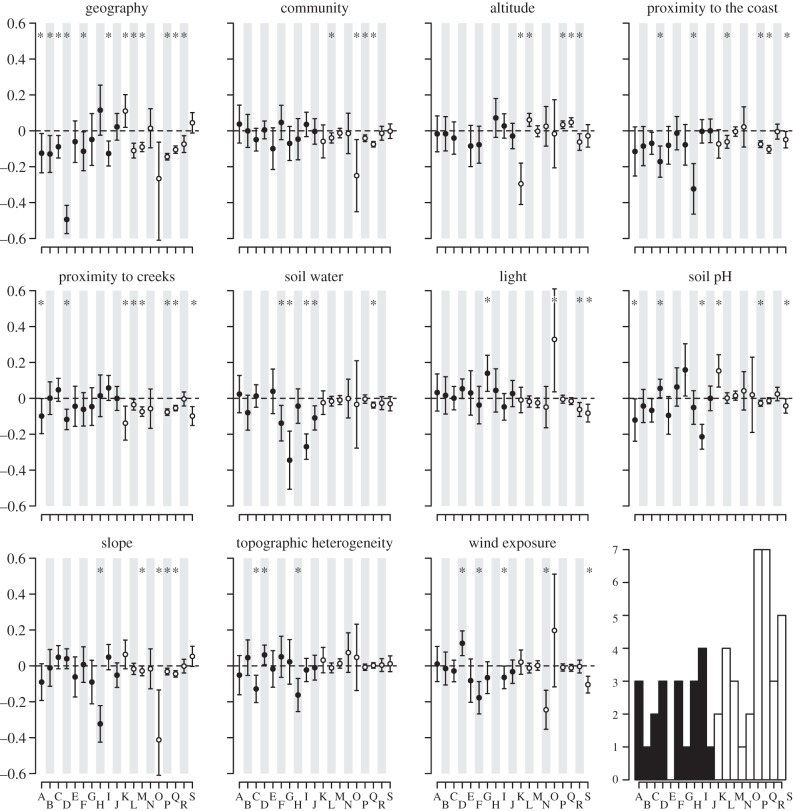
Twelve matrix model averaging results of IBD, IBC and isolation by environmental variables (IBE). Each plot depicts effect sizes and 95% confidence intervals for each species grouped by environmental variable. See figure 1. A*, Alyxia lindii;* B*, Alyxia ruscifolia;* C*, Atractocarpus stipularis;* D*, Coprosma prisca;* E*, Dracopyllum fitzgeraldii;* F*, Geniostoma petiolosum;* G*, Macropiper excelsum* subsp. *psittacorum;* H*, Macropiper hooglandii;* I, *Xylosma maidenii;* J*, Zygogynum howeanum;* K*, Coprosma huttoniana;* L*, Coprosma lanceolaris;* M*, Coprosma putida*-N; N*, Coprosma putida*-S; O*, Coprosma* sp. nov*.;* P*, Howea belmoreana;* Q*, Howea forsteriana;* R*, Metrosideros nervulosa;* S*, Metrosideros sclerocarpa*.

**Figure 3.**
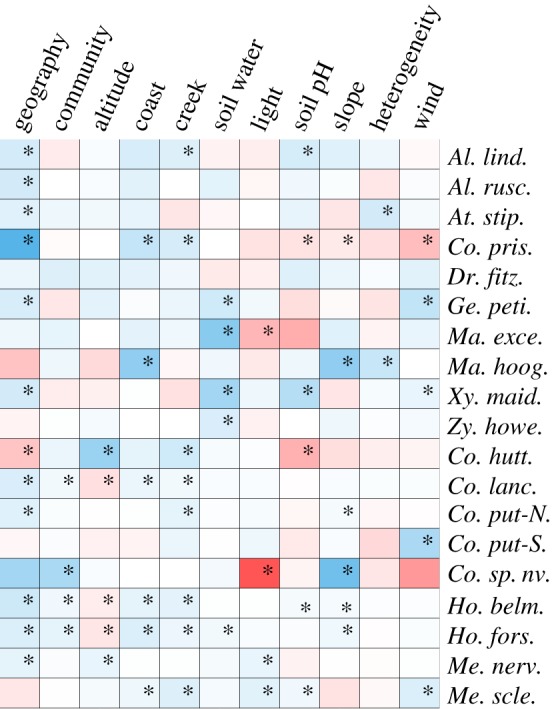
Heat map of IBD, IBC and isolation by environmental variables (IBE). Negative effects in blue, positive effects in red. Asterisk denotes significance (as in figure 1).

The 12 matrix analyses, with each environmental variable included as a separate predictor, produced results broadly consistent with the three matrix analyses. However, there were important differences in some species, and IBE was more widespread (figures 2 and 3). Variance inflation factors of greater than five were detected in four species, and the affected variables were removed from analysis in these species (*C. prisca*—altitude and soil water, *Coprsma* sp. nov*.*—proximity to creeks and the coast, *Macropiper excelsum*—altitude, and *Macropiper hooglandii*—wind exposure). Isolating effects were detected in 18 species, but not in *Dracophyllum fitzgeraldii*. Out of these 18 species, IBD was evident in 11 (six ASG versus four SSG), IBE (isolation by at least one environmental variable) was present in 17 species and IBC in four species (all SSG). Geographical distance was the sole driver of isolation only in *A. ruscifolia*, whereas this was true for environmental variables in five species (three ASG versus two SSG). Significant counter-gradient effects were more common in these analysis; a positive correlation with geography in one species (*C. huttoniana*), and with at least one environmental variable in seven species. Differences between individuals in the distance to the nearest creek was the most common source of isolation owing to any individual variable (present in eight species), followed by differences in proximity to the coast (six species). Counter-gradient effects due to elevation were the most common (three species).

The patterns of IBD, IBE and IBC are consistent between the analyses in 11 of the 19 study species. When environmental variables were distilled into a single measure of environmental dissimilarity, the pronounced differences occurred in: *C. huttoniana*—IBC was gained while strong IBE (altitude and creek) and counter-gradient effects of environment and geography were lost; *D. fitzgeraldii*—no significant correlations were replaced with marginally significant effects of IBD and IBC; *Coprosma* sp. nov*.*—IBE (slope) was replaced by IBD; *C. prisca*, *C. putida*-S and *Z. howeanum*—the IBE effects (coast, wind and soil water, respectively) were lost; *Coprosma lanceolaris*—IBC was lost and in *Atractocarpus stipularis*—IBC was gained.

### (c) Population structure

**Figure 4.**
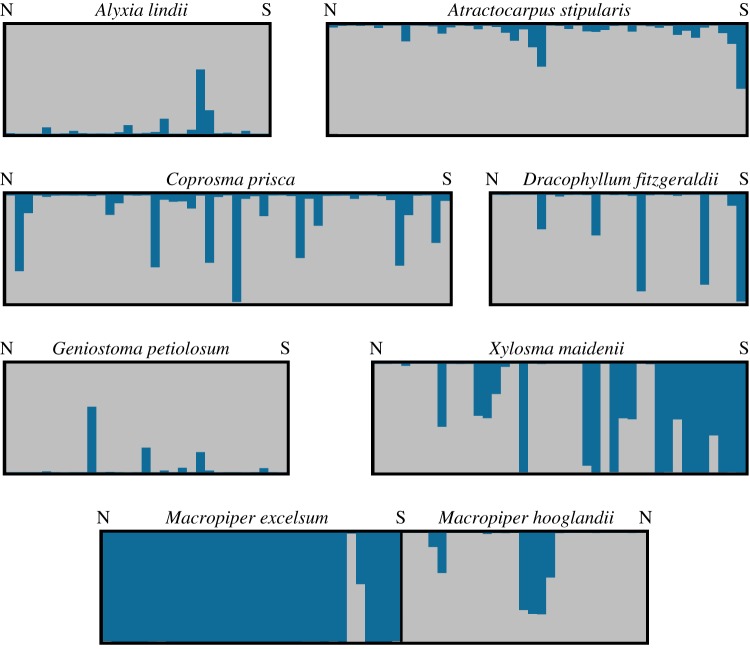
Population genetic structure in the ASG species. Clusters within species were spatially intermixed, except in *X. maidenii*. Each vertical bar represents an individual and colours denote population membership. Individuals are ordered by latitude, N, north, S, south. Each plot depicts a Structure analysis with *K* set to 2.

Previous research has shown that no population structure has been observed within species in the SSG [26]. Here, we analysed the ASG and found no clear population subdivision in *A. ruscifolia*, *Z. howeanum*, *M. excelsum* subsp. *psittacorum* and *M. hooglandii*, although the analyses detected considerable hybridization between the two closely related *Macropiper* species. Statistical assessments indicate that there are two genetic clusters in two widespread endemic species (electronic supplementary material, appendix S2): *Atractocarpus stipularis* and *Xylosma maidenii* (electronic supplementary material, table S1)*.* However, the structure plot does not show clear subdivision in *Atractocarpus stipularis* (figure 4). Based on Δ*K*, *Alyxia lindii, C. prisca*, *D. fitzgeraldii* and *Geniostoma petiolosum* may have two populations present on the island, however the results were not conclusive as only a marginal increase in [Pr(X|*K*)] for *K* = 1 over *K* = 2 was evident. In *X. maidenii*, the populations show some spatial separation along the island's latitudinal gradient, but there was no clear spatial separation of populations within the other species (figure 4).

## 4. Discussion

### (a) Current patterns of isolation on Lord Howe Island

Analysis of the genetic relatedness of individuals from 19 vascular plant species on LHI confirms that environmental and ecological gradients have roles in shaping the patterns of gene flow within this minute island. Environmental dissimilarity, as a single metric or included as specific environmental gradients, contributes to reductions in migration and/or reproduction in as many as 17 species, belonging to a range of plant families and with varying degrees of phylogenetic relatedness. A variety of environmental variables affect genetic structure in the LHI species, with some species under the influence of single gradients and others subjected to many (figures 2 and 3). The contributing factors to isolation within species are often different between sister species and congenerics (e.g. in *Metrosideros*, *Macropiper* and *Coprosma*). This points to the unpredictable and diverse ways in which plants can adapt locally to their environment and is consistent with studies that have shown adaptive responses of multiple plant species to environmental gradients [45,46]. The exact mechanisms that lead to these relationships are less clear. However, natural selection for genotypes with greater fitness in different habitats [1,6,47–49] or habitat induced variation in assortative mating (e.g. through shifts in flowering time) are the most likely drivers of such patterns [50,51]. Whatever the mechanism, it is clear that environmental variation plays a role in shaping the genetic landscape within species even at fine scales.

Despite the small size of the island, IBD was also a common phenomenon, though less widespread than IBE in both assessments. It is clear, therefore, that dispersal limitation can have a significant impact on relatedness within plant taxa with a mixture of dispersal abilities, even when the potential for geographical isolation is severely limited. Although growth form (i.e. plant habit) and dispersal mechanism have been implicated in shaping patterns of spatial genetic structure in plants [8,52,53], these factors have apparently little impact on the observed patterns within LHI species (table 1). These results imply that the small scales at which plants undergo reductions in gene flow and subsequent speciation [54] are not only affected by dispersal limitation, but also reflect the selective process that plant populations are subjected to in spatially structured environments.

Our assessment also included an estimate of isolation due to differences in the local plant community (IBC). These data were extracted from the literature and may be less accurate than direct field observations. Despite this, the occurrence of IBC in several species suggests that this index reflects the importance of ecological relationships and acknowledges that competitive interactions between species can drive divergence [26,55]. Importantly, in the 12 matrix analyses, IBC was only detected in the SSG. Competitive interactions within these genera have been implicated in speciation and the maintenance of species boundaries [26]. This result indicates that IBC may be the extra component of isolation necessary for local adaptation to progress to speciation in sympatry or parapatry.

### (b) How common is isolation by environment?

In general, the high frequency of IBE relationships observed on LHI is consistent with that reported in the literature across taxa [15–18]. Surprisingly, given the known adaptive ability of many plants [45,46], plant studies generally demonstrate lower IBE effect sizes [15], and the majority reject hypotheses of IBE and detect IBD more frequently [18]. Why then is there such a strong indication of IBE on LHI? The island may be unusual in that the highly variable structure of habitat and environments can impose a great diversity of selection pressures. For LHI plants, the range of potential plant stressors, such as restricted light in mountainous areas, salt exposure, variable water availability, and temperature and humidity variability may induce strong selective environments that drive adaptation. Although the island environment is highly heterogeneous, these factors are not specific to LHI. Many oceanic islands possess similar ranges of potential selection pressures and anagenetic evolution of plant species on islands has been shown to decline with increasing heterogeneity [27,56]. Such variability is equally common in continental settings, leading to observations of adaptation to environmental gradients and IBE in a range of continental plant species [18,46]. It may also be possible that plants that have the ability to colonize isolated volcanic islands retain a high diversity of adaptive standing genetic variation [57]. However, there is no direct evidence of this and island populations tend to have lower genetic diversity than continental populations [58]. It is more likely that the relaxed competition afforded by newly emergent islands allows new niches to be exploited by colonizers, free from the crowded and highly competitive communities found in other locations. Through such ecological release, island species may occupy broader ecological niches [59,60]. Alleles that confer local adaptation in an island context may not be sufficient to allow survival in similar, but more competitive, settings, giving rise to the prevalence of IBE on LHI. This is difficult to discount without examination of IBE in species that are distributed both on islands and elsewhere.

Investigation of IBE and IBD across an Antillean community of lizards (*Anolis*) revealed similar variability in both IBD and IBE across different species to that observed in the LHI flora [17]. Converse to our findings, IBD was more common than IBE in *Anolis* species, suggesting that this may not necessarily be a general feature of island taxa. However, the comparison between LHI and the considerably larger Antillean islands should be made with caution. When IBE is tested across scales well beyond the dispersal distance of the organism, gene flow can not only be directly affected by the dissimilarity between the two sampled sites, but must also depend on the intervening habitats. As a result, the individual-based, fine scale patterns observed here may not translate to larger scale, population genetic isolation. Recent reviews that have examined the prevalence of IBE collated data in population-level studies that commonly used *F*_ST_ as a measure of gene flow, rather than the individual-based approach exploited here [15,18]. Scale effects were not evident in these studies, but further examination of IBE and IBD at the individual level is required to establish whether these approaches produce consistent patterns.

### (c) Methodological considerations

Intuitively, the simultaneous evaluation of the isolating effects of multiple variables is an improvement over other methods. Amalgamation of the environmental variables can have an effect in several ways, including the loss of the signal of isolation for specific variables due to confounding effects. Variables that are apparently responsible for the most isolation are not necessarily those that vary the most in the sample, and, as a result, the use of a principal component analysis to generate a dissimilarity matrix may mask the effect of these variables. This is a particularly acute problem for those species in which one strong association or a few weak associations are present (e.g. *Coprosma* sp. nov*.*, *Coprosma putida*-S, *Z. howeanum*). Similarly, combining variables that possess a mixture of isolating influences and counter-gradient patterns into a single measure has the effect of cancelling out any signal (e.g. *C. huttoniana* and *C. prisca*). As an extension of this, failing to estimate effects of individual variables through the use of the single metric can lead to apparently spurious effects of IBD and IBC (*D. fitzgeraldii*, *Coprosma* sp. nov*.*, *Atractocarpus stipularis*). On the other hand, when environmental effects may be individually weak or sources of selection multivariate, combining the environmental variables into one measure of environmental dissimilarity may allow these patterns to be observed. This may explain the discrepancy between the two approaches used in this study, but does not account for the differences in the frequency of IBE in plants found in this study and in the large number of studies that have examined variables separately. Testing multiple variables is much more likely to find correlation than testing only one. Comparison of our results with the assessments of isolation by altitude and pH for the SSG of LHI plants (see [26]) demonstrates that restricting analyses to one or two variables will often miss the pattern that is evident when many are taken into account. After correcting for geographical distance (partial Mantel test), the previous study found some evidence of IBE in both *Metrosideros* species, both *Howea* species and *C. putida*-N. The current analysis showed IBE in all of the *Metrosideros*, *Howea* and *Coprosma* species.

### (d) New insights for speciation on Lord Howe Island

The prevalence of environmental influences on relatedness within taxa supports previous research which concluded that ecological speciation with gene flow may have occurred multiple times on LHI in distantly related taxa (*Howea*, *Metrosideros* and *Coprosma*). Evidence for ecological speciation in these three genera includes (i) divergence without significant geographical isolation, (ii) genetic signatures of divergent selection (detected using outlier analyses), (iii) associations of individual loci with ecological variation (an indication of local adaptation within species), (iv) ecological divergence of species in each genus and (v) competitive exclusion of congeneric species [8,26,28,30]. The implication from the current study—that is, local adaptation within species on LHI is not only possible, but the norm—further enhances the chances that in some taxa this will lead to sufficiently strong reproductive isolation to cause speciation.

Distinct genetic population clusters were detected in one species, and a further four species may also be dividing into genetic clusters, an indication that the flora harbours species at varying stages along the speciation-with-gene-flow continuum [3]. With the exception of *D. fitzgeraldii*, all of these species demonstrated some evidence of both IBD and IBE. The partial spatial separation of populations of *X. maidenii* and presence of IBD in this species do suggest geography has played a role in the reduction of gene flow leading to population divergence. Nevertheless, the populations are divided between the wet south of the island and the dry north leading to strong patterns of IBE, with hybrid individuals in both areas of the island. As a result, the relative influences of IBD and IBE on population divergence remain unclear. However, it is important to note that in all species except for *X. maidenii* the population clusters are spatially intermixed, suggesting that the geographical component of isolation alone is not strong enough to cause divergence. Again, this corroborates data suggesting that pre-zygotic barriers (geographical isolation and flowering time isolation) were not sufficiently strong to cause speciation in *Metrosideros*, *Howea* or the *Coprosma* radiation [26].

IBD and IBE are clearly important phenomena for the flora of LHI. Simultaneously estimating the isolating effects of multiple environmental gradients provides a more detailed understanding of isolating barriers. Our analyses suggest that using this approach in other systems will reveal that IBE is more pervasive than imagined. The apparently widespread occurrence of local adaptation supports a growing body of evidence for the potential for natural selection to overcome the homogenizing influence of gene flow, even at fine scales. Different plant taxa can respond to a variety of selection pressures and in some cases the strength of the ecological isolating barrier can lead to speciation in the same geographical area. Although it is possible that these patterns are unique to the flora of LHI, the growing body of evidence supporting IBE in many taxa and locations suggests that ecologically driven isolation is, indeed, a major force in the accumulation of species diversity.

## Supplementary Material

Supplementary material
