# Supplementary material for: Evaluation of genetic isolation within an island flora reveals unusually widespread local adaptation and supports sympatric speciation
Source: Philos Trans R Soc Lond B Biol Sci. 2014 Aug 5;369(1648):20130342. doi: 10.1098/rstb.2013.0342 (PMC4071517; doi:10.1098/rstb.2013.0342)
Supplement: Supplementary material [file rstb20130342supp1.pdf]

Electronic supplementary material (ESM)

ESM Appendix S1: Primer combinations used for AFLP selective amplification.

Alyxia lindii	*Dracophyllum fitzgeraldii*	*Macropiper hooglandii*
EcoRI-ACT-FAM/MseI-CTC<br>EcoRI-ACA-FAM/MseI-CTG<br>EcoRI-AAG-JOE/MseI-CTT<br>EcoRI-AAC-NED/MseI-CAA	EcoRI-ACT-FAM/MseI-CAT<br>EcoRI-AAG-JOE/MseI-CAC<br>EcoRI-AAG-JOE/MseI-CTT<br>-	EcoRI-ACT-FAM/MseI-CAT<br>EcoRI-AGG-JOE/MseI-CAT<br>EcoRI-AAG-JOE/MseI-CAT<br>-
Alyxia ruscifolia	*Geniostoma petiolosum*	*Xylosma maidenii*
EcoRI-ACT-FAM/MseI-CTC<br>EcoRI-ACA-FAM/MseI-CTG<br>EcoRI-AAG-JOE/MseI-CTT<br>EcoRI-AAC-NED/MseI-CAA	EcoRI-ACA-FAM/MseI-CTG<br>EcoRI-AAG-JOE/MseI-CAC<br>EcoRI-AAG-JOE/MseI-CTC<br>EcoRI-AAC-NED/MseI-CAA	EcoRI-ACT-FAM/MseI-CAT<br>EcoRI-AGG-JOE/MseI-CAT<br>EcoRI-AAG-JOE/MseI-CTT<br>-
Atractocarpus stipularis	*Macropiper excelsum*	*Zygogynum howeanum*
EcoRI-ACT-FAM/MseI-CAC<br>EcoRI-ACA-FAM/MseI-CTG<br>EcoRI-AAG-JOE/MseI-CAC<br>EcoRI-AAG-JOE/MseI-CTT	EcoRI-ACT-FAM/MseI-CAT<br>EcoRI-AGG-JOE/MseI-CAT<br>EcoRI-AGG-JOE/MseI-CTC<br>-	EcoRI-ACT-FAM/MseI-CAT<br>EcoRI-ACT-FAM/MseI-CAC<br>EcoRI-AGG-JOE/MseI-CAT<br>-

ESM Appendix S2. Evaluation of STRUCTURE results and choice of appropriate value of K.

Alyxia lindii

K	Mean Ln[Pr(X│K)]	St.dev Ln[Pr(X│K)]	ΔK
1	-1566.70	5.47	—
2	-1439.36	4.61	28.30
3	-1442.54	11.95	0.06
4	-1445.05	13.19	0.69
5	-1456.71	9.68	1.89
6	-1450.04	11.45	1.47
7	-1460.20	15.20	66.62
8	-2482.88	3208.95	—

Alyxia ruscifolia

K	Mean Ln[Pr(X│K)]	St.dev Ln[Pr(X│K)]	ΔK
1	-1398.21	10.73	—
2	-1428.50	8.65	2.21
3	-1477.93	43.92	1.20
4	-1474.54	45.26	1.22
5	-1416.02	59.03	0.22
6	-1370.27	33.99	3.05
7	-1428.24	167.40	1.17
8	-1682.51	1091.19	—

Atractocarpus stipularis

K	Mean Ln[Pr(X│K)]	St.dev Ln[Pr(X│K)]	ΔK
1	-3511.79	6.06	—
2	-2881.79	7.02	123.28
3	-3117.36	244.48	0.44
4	-3460.79	280.23	0.74
5	-4012.27	626.47	1.21
6	-3805.95	500.47	1.03
7	-4114.95	640.14	0.81
8	-3904.01	203.31	—

Coprosma prisca

K	Mean Ln[Pr(X│K)]	St.dev Ln[Pr(X│K)]	ΔK
1	-2162.53	1.39	—
2	-1978.55	6.00	13.32
3	-1874.50	10.94	11.95
4	-1901.22	150.09	5.35
5	-2731.27	1427.29	1.10
6	-1996.37	441.14	2.21
7	-2238.29	579.61	0.79
8	-2024.62	214.48	—

Dracophyllum fitzgeraldii

K	Mean Ln[Pr(X│K)]	St.dev Ln[Pr(X│K)]	ΔK
1	-2512.32	5.96	—
2	-2297.79	6.15	40.60
3	-2332.83	30.87	12.17
4	-2743.69	358.23	0.48
5	-3324.91	401.91	0.50
6	-3703.80	1733.30	0.45
7	-3309.17	201.96	2.48
8	-3415.03	470.61	—

Geniostoma petiolosum

K	Mean Ln[Pr(X│K)]	St.dev Ln[Pr(X│K)]	ΔK
1	-1511.70	7.02	—
2	-1350.68	12.76	12.49
3	-1349.07	8.21	0.38
4	-1344.32	9.45	184.51
5	-3082.92	4641.15	0.75
6	-1355.74	12.02	144.90
7	-1370.34	10.62	4.99
8	-1437.95	225.91	—

Macropiper excelsum and *Macropiper hooglandii*

K	Mean Ln[Pr(X│K)]	St.dev Ln[Pr(X│K)]	ΔK
1	-2891.74	2.98	—
2	-1034.23	3.27	534.94
3	-924.20	9.82	10.48
4	-917.08	3.20	3.76
5	-921.99	2.77	1.05
6	-923.99	4.59	0.05
7	-926.20	13.58	0.53
8	-935.54	7.38	—

Xylosma maidenii

K	Mean Ln[Pr(X│K)]	St.dev Ln[Pr(X│K)]	ΔK
1	-4655.07	7.41	—
2	-2532.87	5.28	362.83
3	-2325.09	243.72	0.42
4	-2220.08	19.49	18.30
5	-2471.82	152.77	1.37
6	-2514.86	79.47	1.61
7	-2429.71	97.09	1.04
8	-2445.20	80.62	—

Zygogynum howeanum			
K	Mean Ln[Pr(X│K)]	St.dev Ln[Pr(X│K)]	ΔK
1	-8322.03	59.21	—
2	-7739.39	22.46	17.70
3	-7554.23	19.60	14.40
4	-7651.44	163.78	19.61
5	-10960.45	5544.87	0.25
6	-12863.73	3340.08	0.37
7	-16000.59	5582.10	0.95
8	-13847.06	6813.09	—
